# Inflation for Colic

**Published:** 1831

**Authors:** 


### INFLATION FOR COLIC.

Mr. JOHN KING, of Irvine, has promulgated a sovereign remedy for the colic in the last number of our contemporary of Glasgow. It is inflation or blowing up of the intestines, per anum. He conceives that it was a happy thought of those who hit upon this remedy in the hour of danger, after all their other efforts had proved nugatory. "It *paralyses*, as it were, the constricted fibres of the bowels, and may be used in the following cases, if not with complete success, at least with advantage, viz. the various kinds of colic, proceeding from torpidity, spasmodic constriction, viscid meconium in new-born infants, impaction, bezoards and other intestinal concretions, volvulus or intussusceptio, and some cases of *hernia.*" ! This is a very encouraging list, very encouraging indeed. Mr. King relates a case in which inflation proved successful, but we need not do more than allude to it. He secured the bladder of a glyster-bag to the nozzle of the bellows, introduced the pipe into the rectum, and puffed away with the best possible effects. Mr. King conjectures that inflation would have been beneficial in some other cases, but he gives the particulars of no more. He regrets that the remedy should have fallen into disuse; had it been a good one it probably would not have done so. We do not ourselves perceive the advantages of air over fluid as an injection, and many individuals with colic would rather let air out than take it in. We say nothing of the *spectacle* of a learned physican puffing up a person's anus with a pair of bellows. The remedy may be one of great importance for any thing we know to the contrary.

